# Increased Nuclear FOXP2 Is Related to Reduced Neural Stem Cell Number and Increased Neurogenesis in the Dorsal Telencephalon of Embryos of Diabetic Rats through Histamine H_1_ Receptors

**DOI:** 10.3390/cells12030510

**Published:** 2023-02-03

**Authors:** Diana Sarahi De la Merced-García, Ángel Sánchez-Barrera, Juan Hernández-Yonca, Ismael Mancilla, Guadalupe García-López, Néstor Fabián Díaz, Luis Ignacio Terrazas, Anayansi Molina-Hernández

**Affiliations:** 1 Departamento de Fisiología y Desarrollo Celular, Instituto Nacional de Perinatología Isidro Espinosa de los Reyes, Montes Urales 800, Miguel Hidalgo, Ciudad de Mexico 11000, Mexico; 2 Unidad de Biomedicina, Facultad de Estudios Superiores (FES)-Iztacala, Universidad Nacional Autónoma de México (UNAM), Av. de los Barrios, Los Reyes Iztacala, Tlanepantla 54090, Mexico; 3 Departamento de Infectología, Instituto Nacional de Perinatología Isidro Espinosa de los Reyes, Montes Urales 800, Miguel Hidalgo, Ciudad de Mexico 11000, Mexico; 4 Laboratorio Nacional en Salud FES-Iztacala, Universidad Nacional Autónoma de México (UNAM), Av. de los Barrios, Los Reyes Iztacala, Tlanepantla 54090, Mexico

**Keywords:** FOXP2, cortical development, hyperglycemia, neural stem cells, Histamine H_1_-receptor, PKC

## Abstract

Diabetic rat embryos have increased cortical neurogenesis and neuron maturation, and their offspring presented altered neuron polarity, lamination, and diminished neuron excitability. The FOXP2 overexpression results in higher cortical neurogenesis by increasing the transition of radial glia to the intermediate progenitor. Similarly, histamine through H_1_-receptor activation increases cortical neuron differentiation. Indeed, blocking the H_1_-receptor by the systemic administration of chlorpheniramine to diabetic pregnant rats prevents increased neurogenesis. Here, we explore the relationship between the H_1_-receptor and FOXP2 on embryo neurogenesis from diabetic dams. Through qRT-PCR, Western blot, immunohistofluorescence, and flow cytometry, we showed an increased FOXP2 expression and nuclear localization, a reduced Nestin expression and -positive cells number, and a higher PKCα expression in the cortical neuroepithelium of fourteen-day-old embryos from diabetic rats. Interestingly, this scenario was prevented by the chlorpheniramine systemic administration to diabetic pregnant rats at embryo day twelve. These data, together with the bioinformatic analysis, suggest that higher H_1_-receptor activity in embryos under high glucose increases FOXP2 nuclear translocation, presumably through PKCα phosphorylation, impairing the transition of radial glia to intermediate progenitor and increasing neuron differentiation in embryos of diabetic rats.

## 1. Introduction

Corticogenesis is a finetuned process during embryo development originating in the dorsal prosencephalic vesicle, which is colonized by neuroepithelial stem cells to conform a stratified epithelium and the dorsal telencephalon, established by bipolar neural stem cells (NSC), denominated radial glia (RG). NSC are multipotent cells with two main characteristics, self-renewal through symmetric division and the ability to generate specialized cell types of the central nervous system (CNS) through asymmetric or non-proliferative symmetric divisions.

The neurons are the first specialized cells to appear during cortical development, followed by astrocytes and oligodendrocytes. Neurons arise directly from NSC or indirectly through intermediate progenitors (IP) and migrate in an inside-out pattern to constitute the characteristic laminar structure of the cerebral cortex, with the deeper layer neurons born first and the superficial at the end [1,2,3].

FOXP2 is a transcription factor known as a deep-layer cortical marker, implicated in the execution and learning of intraspecies communication, such as language in humans, singing in birds, and ultrasonic vocalization in rodents [4,5,6,7,8,9]. In mice, during CNS development, FOXP2 has been involved in cell proliferation, neurogenesis, migration, and neurite outgrowth and branching [10,11,12]. The knockdown of FOXP2 promotes a neurodevelopment delay, alters neuron differentiation, and impairs ultrasonic vocalization in mice [1,5,6,13,14]. However, its role in NCS proliferation and neurogenesis is controversial. For example, using shRNA to knock down FOXP2 expression, a reduction of RG transition to IPs and reduced neurogenesis was reported [4]. In contrast, *Foxp2* deletion with an early expressing Emx1-cre driver line (E10.5) does not disrupt neurogenesis in the somatosensory cortex [15].

Interestingly, maternal diabetes increases cortical neuron differentiation and maturation, events prevented by the systemic administration of chlorpheniramine (Chlo), an antagonist/inverse agonist of the histamine type 1 receptor (H_1_R). Furthermore, H_1_R is highly expressed in 12-day-old (E12) embryos from diabetic rats [16,17,18], and its activation increase deep-layer cortical neuron differentiation in vivo and in vitro under control conditions [19,20].

Histamine is one of the first neurotransmitters to emerge in the rat CNS [21], which acts as a neurogenic factor in the developing brain through the H_1_R [20,22]. The H_1_R interacts with Gα_q/11_ proteins to activate phospholipase C (PLC) and the production of two-second massagers, inositol 1,4,5-trisphosphate (IP3) and diacylglycerol, which in turn promotes IP3-dependent release of Ca^2+^ ions from intracellular stores and the activation of classical protein kinase C, particularly the isoenzyme alfa (α) [21].

The similarities between the presumptive role of FOXP2 and the H_1_R effect on cortical neuron differentiation suggest that this transcription factor on the increased cortical neurogenesis of embryos from diabetic rats might be regulated through the H_1_R signaling pathway in such a way that it could change FOXP2 expression or its subcellular localization in NSC.

Here, we explore FOXP2 participation in neurogenesis and its relationship with the higher H_1_R activity in the cortical neuroepithelium of embryos from diabetic rats. We first corroborated the effect of maternal diabetes and the systemic administration of Chlo on neuronal markers at E14. Then, through molecular, biochemical, and bioinformatic approaches, we evaluated the maternal diabetes effects and the systemic administration of Chlo to diabetic rats on FOXP2 expression and intracellular localization; Nestin and TBR2 cells; PKCs expression; PKCα activity, as well the potential PKC phosphorylation in FOXP2^+^-cells.

## 2. Materials and Methods

### 2.1. Animals

Female Wistar rats (*Rattus Norvegicus*; 250–300 g) from the “Instituto Nacional de Perinatología Isidro Espinosa de los Reyes” animal facilities were maintained under standard conditions with access to water and food ad libitum [18]. The morning after mating, a vaginal smear was performed to confirm the presence of spermatozoids, and this time point was determined as E0.5.

Pregnant rats were aleatorily distributed into two groups at E5. The control (Ctl) was intraperitoneally injected with citrate buffer (0.1 M, pH 7.4), while the diabetic (Db) with 50 mg/Kg streptozotocin (Sigma-Aldrich^®^, Saint Louis, MO, USA) [18]. Forty-eight hours later, the glycemic levels were measured using an electronic glucometer (Accuchek-ROCHE, Basel, Switzerland). Rats injected with streptozotocin with glucose levels higher than 200 mg/dL were assigned to the Db group. Animals that received a citrate-buffered solution with 90–115 mg/dL of glucose were included in the Ctl group [23]. At E12, the Db group was subdivided into a new Chlo-treated diabetic group (Db + Chlo; Sigma-Aldrich^®^) for three groups of pregnant rats (Figure S1A).

Pregnant rats were decapitated at E14, and the embryos were rapidly removed by cesarean and placed in cold Krebs solution (NaCl 100 mM, KCl 2 mM, KH_2_PO_4_ 0.6 mM, NaHCO_3_ 12 mM, glucose seven mM, 0.1% phenol red, 0.3% albumin from bovine serum, and 0.3% de MgSO_4_). Only embryos without neural tube defects were used in this study (Figure S1B–E).

Two to four embryos per litter from 3–5 pregnant rats per group were fixed by immersion in Boüin’s solution for 24 h, washed in phosphate buffer saline pH 7.4 (PBS), and placed in sucrose gradients (15 and 30%) for 24 h each and maintained for immunohistofluorescence (Figure S1A). The remaining embryos were dissected to obtain the dorsal telencephalon (cortical neuroepithelia) under a stereoscopic microscope (Olympus Corporation S7X2-ILLT, Tokyo, JPN) and processed for flow cytometry or maintained frozen until used (−80 °C) for qRT-PCR and Western blot (Figure S1A).

### 2.2. Quantitative Reverse-Transcription Polymerase Chain Reaction (qRT-PCR)

**Figure 1:**
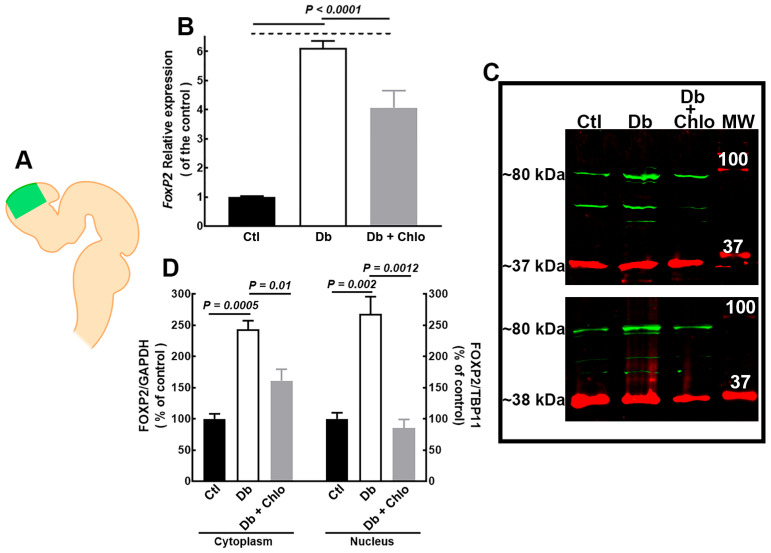
**FOXP2 expression in the cortical neuroepithelium at 14-day-old embryos.** (**A**) Image showing the E14 neural tube. In green is highlighted the dorsal telencephalon dissected for qRT-PCR and Western blot analysis. Created with BioRender.com (1 January 2023). (**B**) FoxP2 expression in E14 dorsal telencephalon from control (Ctl), diabetic (Db), and Chlorpheniramine-treated diabetic (Db + Chlo) groups. Gapdh amplification was used as an internal control. Values (means ± S.E.M., *n* = 5) are expressed as the relative expression of the control using the 2^−ΔΔCT^ method. (**C**) Representative FOXP2 (green, ~80 kDa) Western blots of cytoplasmic (up) and nuclear (down) protein extracts. GAPDH (red, ~37 kDa) and TBP1 (~38 kDa) were used as internal controls for cytoplasm and nuclear fractions, respectively. (**D**) Quantitative fluorometry analysis of FOXP2. Values (means ± S.E.M., *n* = 4) are expressed as a percentage of the control fluorescence ratio. The two-way ANOVA, followed by Tukey’s multiple comparisons test, was performed, and the significant *P* values are shown in the graphs. MW = molecular weight ladder.

The total RNA from a pool of six E14 dorsal telencephalons per experiment (Figure 1A; *n* = 5) per group was isolated using TRIZOL reagent (ThermoFisher Scientific, Waltham, MA, USA). The RNA integrity was determined by visualizing 18S and 28S ribosomal RNA bands stained with GelRed (0.2 mg/mL; Biotium, San Francisco, CA, USA) in 2% agarose gel. One microgram of RNA was used for the reverse-transcription reactions following the protocol recommended by the provider (Promega, Madison, WI, USA).

*Map2*, *β-III Tubulin*, and *Gapdh* primers (Supplementary Table S1) were previously validated by Solís et al. (2017), whereas *Foxp2*, *PKCα*, *PKCβ*, and *PKCγ* PCR products, were recovered from agarose gels using the Zymoclean^TM^ Gel DNA Recovery Kit (Zymo Research, Irvine, CA, USA) and sequenced in the Molecular Biology Unit-IFC-UNAM. Using BLAST^®^ (standard nucleotide BLAST), a 100% identity was obtained for each product corresponding to nucleotides: 102 to 323 of NM_001271104.1, 790–1000 of NM_001105713.1, 1484–1282 of NM_012713.4, and 215–438 of NM_02628.2 for *Foxp2*, *PKCα*, *PKCβ*, and *PKCγ*, respectively.

Dynamic ranges were performed using a Rotor-Gene thermocycler (QIAGEN, Venlo, NLD) to obtain the PCR efficiency and threshold cycle values for each PCR product. After each reaction, melting curves were performed to ensure a single amplified product. PCRs were performed with 500 ng of cDNA and the KAPA SYBR^®^ FAST qPCR Master Mix 2X (KAPA Biosystems, Wilmington, MA, USA) containing 0.8 pmol of each sense and anti-sense primers (Supplementary Table S1) in a final volume of 10 μL. The PCRs conditions were as follows: 10 min denaturalization at 95 °C followed by 35 cycles of denaturalization (30 s at 95 °C), aligning (15 s; for temperatures and primer sequences, see Table S1), and extension (30 s at 72 °C). The *Gapdh* amplification was used as internal control, whereas total RNA and the PCR mix without cDNA were negative controls. The method 2^−ΔΔCT^ was used to evaluate changes in the relative expression between groups [24].

### 2.3. Immunohistofluorescence

Two fixed embryos per experiment were frozen by isopentane immersion (−80 °C) and embedded in Tissue-Tek (Sakura Finetek USA, Inc., Torrance, CA, USA) for a total of 3–5 experiments per group.

**Figure 2:**
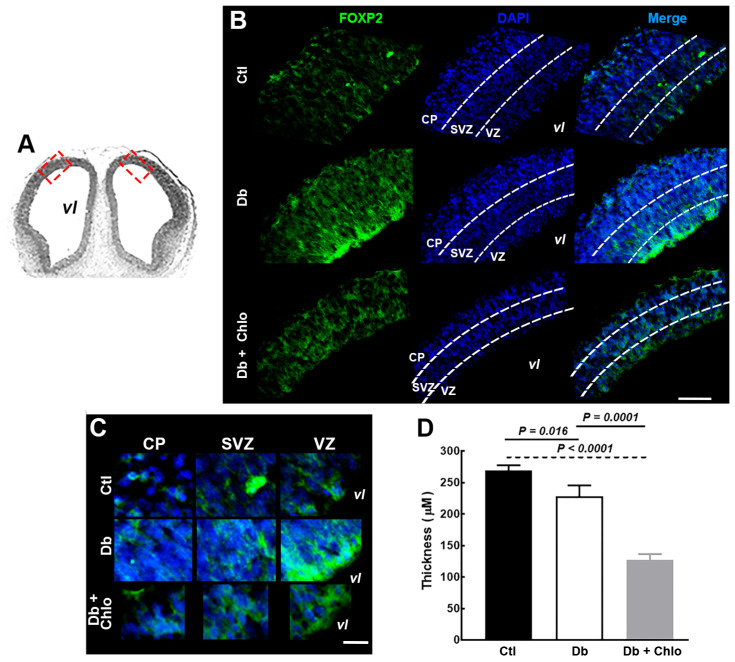
**FOXP2 distribution in the cortical neuroepithelium at 14-day-old embryos.** (**A**) Hematoxylin-eosin-stained coronal section of E14 telencephalon used for the immunodetection in (**B**) and (**C**). vl = ventricular lumen. The red dotted line rectangle represents the zone of the cortical neuroepithelium where micrographs were taken (bilateral). (**B**) Representative micrographs (20×) of FOXP2 signal (green) of E14 coronal sections from control (Ctl), diabetic (Db), and chlorpheniramine-treated diabetic (Db + Chlo) groups. (**C**) Electronic zoom (300%) of the FOXP2 (green) mark in the cortical plate (CP), the subventricular zone (SVZ), and the ventricular zone (VZ). For all images, nuclei are stained with DAPI (blue) and represent 3–4 independent experiments with four determinations. Scale bar = 100 µm and 25 µm for (**B**) and (**C**), respectively. (**D**) Analysis of the cortical neuroepithelium thickness. Data were obtained from 3 measurements per slice (3 slices) and are the mean ± S.E.M of 3–4 independent experiments. The two-way ANOVA, followed by Tukey’s multiple comparisons test, was performed, and the significant *P* values are shown in the graphs.

Coronal sections (10 μm thick; Figure 2A) containing the frontal telencephalon were obtained using a cryostat (Leica CM1850 UV, Leica Biosystems, Wetzlar, DEU), and six consecutive slices were placed per poly-L-lysine coated slides. The tissue was blocked, permeated (10% normal goat serum and 0.3% Triton-X100 in PBS, pH 7.4), and incubated at 4 °C overnight with primary antibodies (Table S2), followed by the fluorescent secondary antibodies (Table S3) for one h at room temperature. At least four consecutive slices were used per antibody. Primary antibody incubation was omitted to create a negative control. Nuclei were stained with 4′,6-diamidino-2-phenylindole (DAPI, one ng/mL; Sigma-Aldrich).

Dorsal telencephalon microphotographs from the frontal epithelium were obtained using an epifluorescence microscope (Olympus IX81) with a charge-coupled device camera (Hamamatsu, ORCA-Flash 2.8, Hamamatsu, JPN). Also, through confocal microscopy (Leica TCS-SP8 DM6000, objective 40× (N.A. 1.3)), the co-localization of FOXP2 with Ki67 or phosphorylated PCKα (PKCα^ph^) was evaluated.

To determine Nestin and TBR2 protein levels in the frontal telencephalon, quantitative immunofluorescence was performed in tissue sections using infrared-coupled secondary antibodies (Supplementary Table S3). An infrared Odyssey CLx (LI-COR, Lincoln, NE, USA) imaging system was used to scan the slides containing six consecutive coronal sections. The Nestin and TBR2 signals were obtained from 4 independent experiments using the Image Studiover.4.0 software (LI-COR). The fluorescent signal was normalized using DRAQ5TM (internal control; 5 μM; Abcam, Cambridge, UK), and the results were expressed as a percentage of the Ctl group.

Representative images were processed using Adobe Photoshop CS6 (Adobe Inc., San Jose, CA, USA).

### 2.4. Western Blot

A dorsal telencephalon pool from three (Ctl) or four (Db and Db + Chlo) litters for three to four independent experiments was obtained (9–16 pregnant rats). Cytoplasmic and nuclear protein extracts were obtained using standard protocols. First, the tissue was homogenated using a POLYTRON^®^ PT 2100 (KINEMATICA AG, Lucerne, CHE) in lysis buffer A containing HEPES 20 mM, MgCl_2_ 1.5 mM, KCl 10 mM, DTT 1 mM, and protease, and phosphatase inhibitors (AMRESCO, Solon, OH, USA). After centrifugation (10 min at 2300× *g*), the supernatant containing the cytoplasmic protein fraction was aliquoted. Next, the pellet was resuspended in a proportion 1:1 of buffers A and B (HEPES 20 mM, MgCl_2_ 1.5 mM, EGTA 0.2 mM, Glycerol 20%, KCl 0.42 mM, DTT 1 mM, protease, and phosphatase inhibitors), incubated during 30 min on ice, and further centrifugated (20 min at 18,000× *g*) to obtain the nuclear fraction. Samples were maintained at −80 °C until used.

The protein concentration was determined by the Bradford method [25]. First, protein electrophoresis was performed in denaturalizing 10 (Nestin) or 7.5% (Map2) polyacrylamide gels using the MiniProtean II system (Bio-Rad, Hercules, CA, USA). Then, proteins were transferred to nitrocellulose membranes (AmershamTM Hybond TM-ECL, Buckinghamshire, UK) using the Trans-Blot semi-dry transfer cell system (Bio-Rad), as described previously [26].

The membranes were blocked with a commercial TBS blocking buffer (LI-COR) and incubated with the corresponding primary antibodies (Supplementary Table S2) diluted in TBS-Tween 0.1% overnight. After removing the non-binding antibodies, the membranes were incubated with infrared secondary antibodies diluted in TBS-Tween 0.1% for one h (Table S3), scanned, and the fluorescence between groups was evaluated using the Image Studiover.4.0 software (LI-COR). The GAPDH (cytoplasmic) or TBP1 (nuclear) proteins were used as internal controls.

### 2.5. Flow Cytometry

New cells from E14 dorsal telencephalons (one litter) for a total of 4–5 experiments per group were mechanically dissociated to a single cell suspension in cold PBS, fixed, and permeabilized using a fluorescence-activated cell sorting (FACS) solution (BD Bioscience, Franklin Lakes, NJ, USA). A fraction containing ~106 cells was incubated with Nestin antibody (Table S2) for 30 min at 4 °C, followed by 30 min incubation with a secondary antibody (Table S3) at 4 °C. Cells without primary or secondary antibodies were used as background fluorescent signal controls. Flow cytometry analysis was performed using a FACS Aria III and the BD FACS Diva software 6.0 (BD Bioscience).

### 2.6. Bioinformatic

The prediction of conventional PKC subfamily phosphorylation sites in FoxP2 (NP_001258033.1) was performed with GPS 3.0-Species-Specific (*R. norvegicous*) using a high threshold cut (false positive rate of 2%), where the score value is proportional to the phosphorylation probability [27]. A second analysis of the phosphorylation sites was performed using the NetPhos-3.1 [28]. The protein phosphorylation site residues were obtained with DOG 2.0 software [29].

The protein secondary structure, relative solvent accessibility, and disorder level were obtained by NetSurfP-2.0 [30], introducing the rat FOXP2 sequence.

### 2.7. Statistics Analysis and Graphs

One one-way ANOVA, followed by Tukey’s multiple comparison test, was performed for comparison between groups. *P* values below 0.05 were considered significant. Statistical analysis and graphic creation were performed in GraphPad Prism version 7.04 (GraphPad Software, Inc., San Diego, CA, USA).

## 3. Results

As expected, significantly less viable embryos were obtained in the Db and Db + Chlo groups. After removing the embryos with neural tube defects and no heartbeat, the viability percentages were 53.8 ± 6.2 and 69.0 ± 6.7 in the Db and Db + Chlo groups, respectively. However, embryos from Db + Chlo rats showed higher viability than the Db group (Figure S1B–E).

An increase in the expression of βIII-Tubulin (βIII-Tub, 1.9-fold) and Map2 (4.5-fold) was observed in the Db group compared to the Ctl, as previously reported [17]. Furthermore, the systemic administration of Chlo at E12 to diabetic rats showed a decreasing βIII-Tub and Map2 compared to the Ctl group (Figure S2B).

Similarly, the Western blot analysis showed a significant increase in βIII-TUB (4.1-fold) and MAP2 (2.9- and 6.0-fold MAP2 a/b and MAP2 c, respectively) in the Db compared to the Ctl. The H_1_R antagonist/inverse agonist again hindered the increased βIII-TUB and MAP2 a/b/c protein levels in the Db group (Figure S2C–F).

As MAP2 c is preferentially expressed during embryo development and MAP2 a/b in the adult brain [31], we calculate each group’s MAP2 c and MAP2 a/b isoforms rate. The resulting rate showed a higher proportion of MAP2c in all groups, with values of 2.4, 4.8, and 2.8 in the Ctl, Db, and Db + Chlo groups (data not shown), respectively.

Finally, it is important to highlight that a Chlo treatment did not affect βIII-TUB and MAP2 isoforms compared to the Ctl (Figure S3A,B).

### 3.1. Effect of High Glucose and Chlorpheniramine on FOXP2 Expression and Subcellular Localization

It has been suggested that FOXP2 expression in the ventricular zone (VZ) is essential for RG transition to IPs and neurogenesis [1], and due to that, Chlo prevents the increased expression of the neuron markers in the dorsal telencephalon in the Db group, we evaluate the expression and cellular localization of FOXP2.

The qRT-PCR analysis showed a higher Foxp2 expression (6.1-fold) in embryos of Db rats as compared with the Ctl group at E14; this effect was partially prevented by Chlo (Figure 1B). Interestingly, the Western blot analysis revealed a higher cytoplasmic (2.4-fold) and nuclear (2.6-fold) FOXP2 protein in the Db than in the Ctl group. Chlo administration to pregnant diabetic rats at E12 ultimately blocked the increased nuclear protein content but did not abolish the cytoplastic (Figure 1C,D). Embryos from Chlo-treated rats did not show changes in nuclear FOXP2 content (Figure S3C).

The immunohistofluorescence showed an atypical FOXP2 mark distributed along the cortical neuroepithelium in all groups. However, an intense signal in the ventricle (VZ) and subventricular (SVZ) zones in embryos from the Db group was observed as compared with the Ctl group (Figure 2A–C). The H_1_R antagonist/inverse agonist systemic administration in the Db group prevented the increase in FOXP2 signal and promoted a significantly thinner neuroepithelium compared to the Ctl group at E14 (Figure 2D).

**Figure 3:**
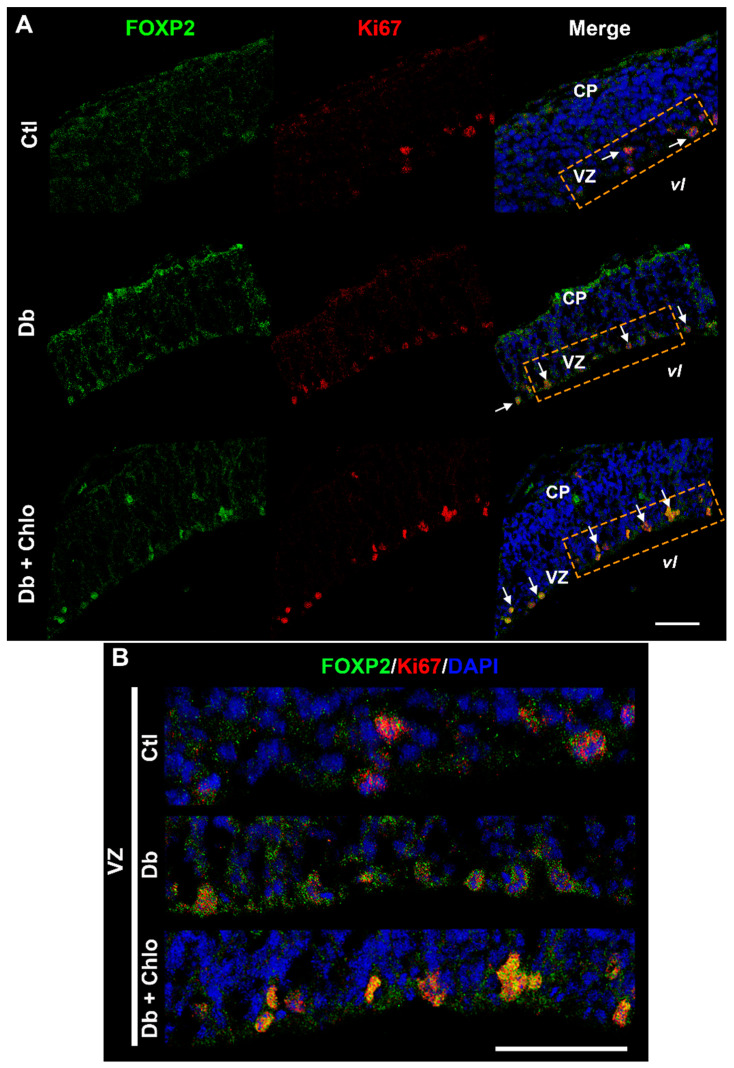
**FOXP2 and Ki67 co-localization in the cortical neuroepithelium at 14-day-old embryos.** (**A**) Single and merge channels from representative confocal micrographs (40×) of FOXP2 (green), Ki67 (red), and nuclei stained with DAPI (blue) taken from the dorsal telencephalon of E14 of control (Ctl), diabetic (Db), and chlorpheniramine-treated diabetic (Db + Chlo) groups (arrows are FOXP2 and Ki67-positive cells). (**B**) Electronic zoom (300%) from the ventricular zone (VZ) of the orange dotted line rectangle in the merge images in (**A**). Scale bars in (**A**) and (**B**) = 50 µm, VZ = ventricular zone, and vl = ventricular lumen.

To confirm the FOXP2 cytoplasmic localization during cortical development, micrographs from the ganglionic eminence (ventral telencephalon) were analyzed. Interestingly, the cytoplasmic staining was scarce in the ventricular zone. In contrast, we found a strong nuclear mark in the distal zone (Figure S4A,B), suggesting that FOXP2 cytoplasmic aggregation could be a cerebral cortex peculiarity. Indeed, cytoplasmic and nuclear marks also were detected by confocal microscopy (Figure 3).

To determine if FOXP2-positive cells display an active cell cycle, we performed FOXP2 and Ki67 double immunohistofluorescences. Both markers were co-expressed in all experimental groups at the VZ (Figure 3). Indeed, FOXP2 staining can be observed in the periphery and into the nuclei, supporting the Western blot data.

### 3.2. Effect of High Glucose and Chlorpheniramine on Neural Stem and Intermediate Progenitor Cells

The FOXP2 expression and their subcellular and tissue localization suggest its possible participation in the increased neurogenesis, probably through a higher transition of RG to IPs, in the Db group. Hence, we investigated Nestin protein level, distribution, and cell number in our experimental conditions.

**Figure 4:**
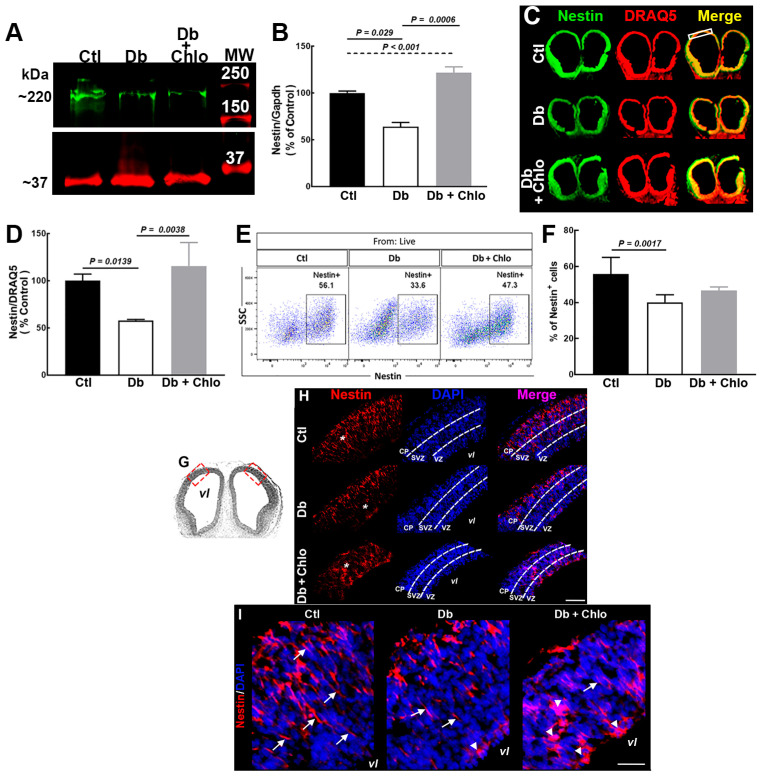
**Nestin positive cell analysis in the cortical neuroepithelium and of 14-day-old embryos.** (**A**) Representative Nestin (radial glia, green, ~220 kDa) Western blots of control (Ctl), diabetic (Db), and chlorpheniramine-treated diabetic (Db + Chlo) groups of E14 dorsal telencephalic tissue. GAPDH (red, ~37 kDa) was used as an internal control. (**B**) Quantitative fluorometry analysis of Nestin. Values (means ± S.E.M., *n* = 4) are expressed as a percentage of the control fluorescence ratio. (**C**) Nestin (green) representative immunofluorescence images of coronal sections from Ctl, Db, and Db + Chlo of E14 coronal sections. DRAQ5 (red) staining was used as the internal control. (**D**) Quantitative fluorometry analysis for Nestin in the dorsal telencephalon (white rectangle in (**C**)). Values (means ± S.E.M., *n* = 4) are expressed as a percentage of the fluorescence ratio of controls. (**E**) Nestin^+^ cells representatives flow cytometry in cells obtained at E14 from the dorsal telencephalon of Ctl, Db, and Db + Chlo groups. (**F**) Quantitative analysis of Nestin^+^ cells. Values (means ± S.E.M., *n* = 4) are expressed as the percentage of the total positive cells. For (**B**,**D**,**F**), the two-way ANOVA, followed by Tukey’s multiple comparison test was performed, and the significant *P* values are shown in the graphs. (**G**) Hematoxylin-eosin-stained coronal section of E14 telencephalon represents the cut level used in the immunodetection. (**H**) Representative micrographs (20×) of Nestin mark (red) and DAPI stained nuclei (blue) from E14 coronal slices of control (Ctl), diabetic (Db), and chlorpheniramine-treated diabetic (Db + Chlo) groups. Cortical plate = CP, subventricular zone = SVZ, ventricular zone = VZ, and vl = ventricular lumen. Scale bar = 100 µm in H and 50 μm in (**I**). (**I**) Electronic zoom (300%) of the micrographs in (**H**) from the zone marked with the white asterisk. The white arrows show Nestin projections and arrowheads aberrant Nestin aggregation.

The Western blot revealed a diminished Nestin content in the Db group compared to the Ctl. Lower Nestin was prevented by Chlo (Figure 4A,B). The above was corroborated by quantitative immunohistofluorescence, which revealed a minor Nestin-positive signal in the Db compared with the Ctl group, an effect also blocked by Chlo (Figure 4C,D). A similar result was obtained by flow cytometry; nevertheless, Chlo in the Db group did not affect the altered cell number of Nestin-positive cells (Figure 4E,F).

The Nestin immunohistofluorescence performed at the same level as in Figure 2A showed the expected continuous Nestin scaffold from the apical to basal cortical neuroepithelium in the Ctl group. In contrast, the staining presented a discontinuous pattern along the neuroepithelium, an aberrant reservoir in the VZ, and a reduction in the SVZ prolongations in the Db group. Unexpectedly, the aberrant Nestin mark was accentuated after Chlo treatment (Figure 4G–I).

**Figure 5:**
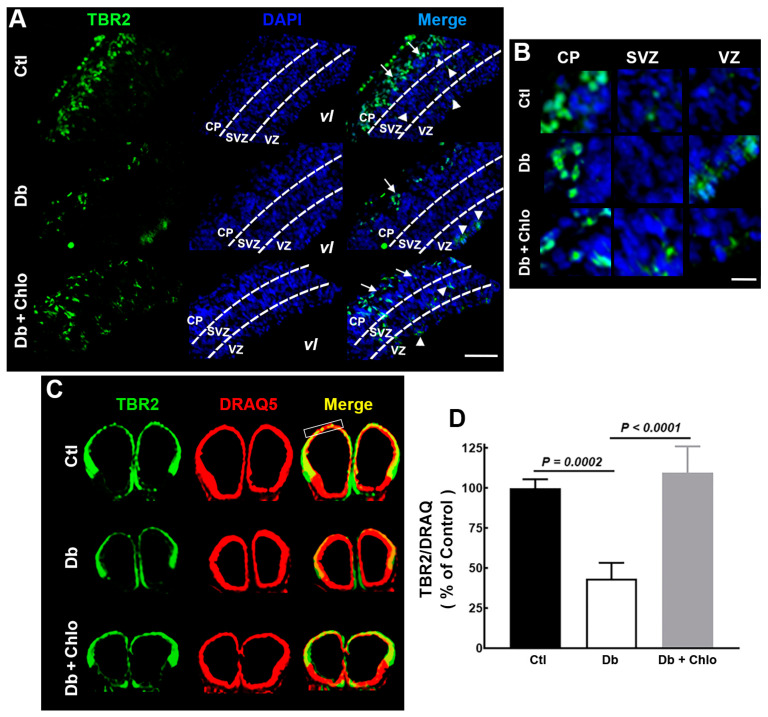
**TBR2 analysis in the cortical neuroepithelium at 14-day-old embryos.** (**A**) Representative Tbr2 (green) epifluorescence micrographs (20×) of coronal sections from control (Ctl), diabetic (Db), and chlorpheniramine-treated diabetic (Db + Chlo) groups at E14. (**B**) Electronic zoom (300%) of the TBR2 (green) mark in the cortical plate (CP), the subventricular zone (SVZ), and the ventricular zone (VZ). For all images, nuclei are stained with DAPI (blue) and represent 3–4 independent experiments with four determinations. Scale bar = 100 µm and 25 µm for (**A**,**B**), respectively. (**C**) TBR2 (green) representative immunofluorescence images of coronal sections from Ctl, Db, and Db + Chlo groups at E14. DRAQ5 (red) staining was used as the internal control. (**D**) Quantitative fluorometry analysis for Tbr2 in the dorsal telencephalon (white rectangle in (**C**)). Values (means ± S.E.M., *n* = 4) are expressed as a percentage of the fluorescence ratio of controls. The two-way ANOVA, followed by Tukey’s multiple comparisons test was performed, and the significant *P* values are shown in the graphs.

NSC transition to IP was evaluated by TBR2 immunohistofluorescence. Unexpectedly, we found minor TBR-positive cells in the Db group compared with the Ctl group, an outcome prevented by Chlo treatment (Figure 5A,B). Accordingly, we detected a lower fluorometric TBR2 signal in the Db group than in the Ctl and Db + Chlo groups (Figure 5C,D).

Furthermore, TBR2-positive cells were mainly observed in the CP and SVZ in the Ctl group. In contrast, IPs were in the CP and VZ in the Db group and along the cortical epithelium in the Db + Chlo (Figure 5A,B). Differences in the TBR2 staining pattern between groups could be associated with impaired migration in the experimental groups, an idea supported by an aberrant Nestin mark in the Db and Db + Chlo groups (Figure 4I).

### 3.3. H_1_R Signaling Pathway Could Be Responsible for the Increased FOXP2 Nuclear Translocation

The signal transduction pathway downstream of H_1_R involves phospholipase C activation promoting IP3 and DAG formation, and consequently classical PKC activation, which can phosphorylate several proteins affecting cell physiology and gene expression. In addition, phosphorylation promotes nuclear protein localization [32]. As a first approach, we demonstrate, in Ctl and Db groups, the presence of the H_1_R in the cortical neuroepithelium in the VZ at E12 (Figure S5).

**Figure 6:**
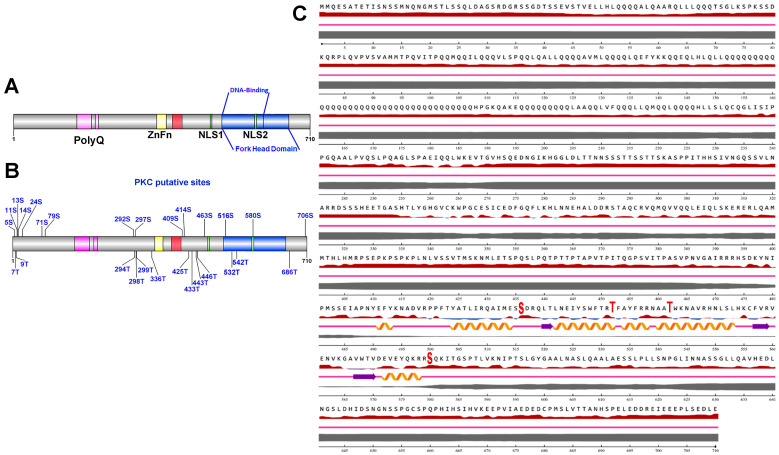
**Bioinformatic analysis of rat FOXP2 PKC phosphorylation sites.** (**A**) Map of FOXP2 protein domains (NP_001258033.1): pink = polyglutamine (Poly-Q), yellow = zinc finger (ZnFn), red = leucine zipper, green = nuclear localization signal 1 and 2 (NLS1 and NSL2) and blue = Forkhead. (**B**) Scheme showing putative PKC phosphorylation sites in FOXP2. (**C**) Sequence and secondary structure of FOXP2 obtained by NetSurfP-2.0. 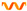 Helix, 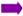 Strand, and 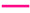 Coil. The relative surface accessibility is represented by the red (exposed sites) and blue (buried sites), and the percentage of the disorder is represented by the gray line, where the thickness is directly related to the level of disorder of each residue.

Then, we performed a bioinformatic analysis that revealed 29 putative PKC phosphorylation sites in the FoxP2 sequence with nine residues shared by the two software algorithms (Figure 6A–C; Table S4). Interestingly, from the shared amino acid residues, the S580 belongs to the second nuclear localization sequence (NLS2; KRRSQK) within the Forkhead domain (Figure 6A,B). Furthermore, the relative surface accessibility (RSA) and PKC putative site disorder level analysis revealed that four amino acids within the Forkhead domain showed medium RSA and low disorder levels (S516, S580, T532, and T542). In addition, S516 and S580 possessed higher relative surface accessibility (Table S4 and Figure 6C).

**Figure 7:**
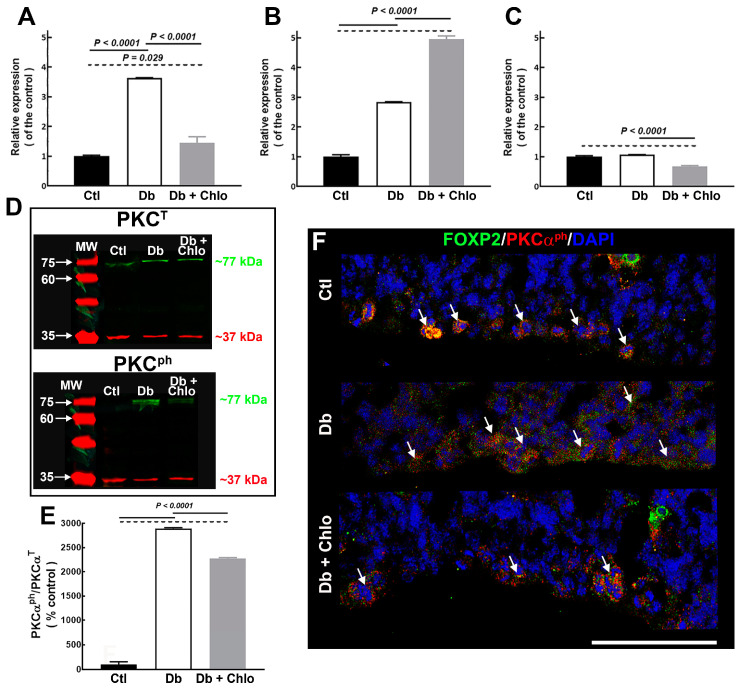
**PKCs expression in the cortical neuroepithelium at 14-day-old embryos.** (**A**–**C**) Graphs of the expression analysis of PKCα (**A**), PKCβ (**B**), and PKCγ (**C**) in the dorsal telencephalon from control (Ctl), diabetic (Db), and chlorpheniramine-treated diabetic (Db + Chlo) groups at E14. Gapdh amplification was used as internal control. Values (means ± S.E.M., *n* = 5) are expressed as the relative expression of the control using the 2^−ΔΔCT^ method. The two-way ANOVA was performed, followed by Tukey’s multiple comparisons test. The significant *P* values are shown in the graphs. (**D**) Representative total and phosphorylated (ph) PKCα (green, ~77 kDa) Western blots from E14 telencephalic tissue of Ctl, Db, and Db + Chlo groups. GAPDH (red, ~37 kDa) was used as a loading control. MW = molecular weight ladder. (**E**) Quantitative fluorometry analysis of PKCα^ph^. Values (means ± S.E.M., *n* = 4–5) are expressed as the rate of the normalized fluorescent values of PKCα^ph^/PKCα^T^ (total PKC). The two-way ANOVA was performed, followed by Tukey’s multiple comparisons test. The significant *P* values are shown in the graphs. (**F**) Confocal images (40×) of the co-localization of FOXP2 (green) and PKCα^ph^ (red). Nuclei are stained with DAPI (blue). Arrows are FOXP2 and PKCα^ph^-positive cells). Scale bar = 100 μm.

To evaluate possible changes in classical PKCs expression, qRT-PCR was performed for the three isoenzymes (α, β, and γ). The results showed a significant increase of PCKα (3.6-fold) and PKCβ (2.8-fold) in the Db compared to the Ctl group. However, Chlo significantly prevented only the rise of PKCα in the Db (1.4-fold) versus the Ctl group (Figure 7A–C). Congruent with the qRT-PCR data, total PCKα and PCKα^ph^ showed a higher protein content in the Db versus the Ctl group (Figure 7D,E). Interestingly, active PCKα (PCKα^ph^) was observed by confocal microscopy in control and experimental groups in the VZ, demonstrating the PCKα^ph^ and FOXP2 co-localization (Figure 7F).

## 4. Discussion

We previously reported that histamine promotes neuron differentiation of cortical NSC through H_1_R activation [22], and the in utero and systemic administration of the antagonist/inverse agonist of the H_1_R, Chlo, decreases neuron differentiation in embryos from control and diabetic rats, respectively [18,19].

Chlo is distributed rapidly and extensively in several body tissues, showing higher concentration in tissues than in the plasma [33,34,35] due to its high passive membrane permeability and moderate protein binding [36,37]. Although direct evidence of Chlo crossing the fetus’s placental barrier has not been reported, we can postulate that the drug reaches the embryo due to the mentioned characteristics and the effect reported here and in other studies [18,38,39]. However, further studies are needed to resolve this issue.

As a first approach, the increased cortical neurogenesis and its prevention by Chlo were corroborated [17] (Figure S2), supporting the H_1_R participation. Moreover, although premature neuronal maturation could be suggested by a higher proportion of the MAP2 a/b isoform in the Db group, it is worth mentioning that MAP2 c was the most abundant variant in the dorsal telencephalon of all groups at E14. Changes in MAP2 isoforms can be explained by mRNA alternative splicing disruption. An idea supported by studies reporting impaired alternative splicing in diabetic animal and human models [40,41,42].

The above suggests that in the embryos of the diabetes model used here, the increased signaling through H_1_R involves a mechanism related to NSC premature differentiation. Interestingly, the activation of this receptor increases FOXP2^+^ cells in cortical NSC in vitro [20]. Moreover, FOXP2 overexpression promotes increased neurogenesis through IPs production and reduced NSC population during cortical development [4].

The cortical neuroepithelium of E12 from diabetic rats at E12 showed higher H_1_R expression than in later embryonic days and control embryos at E12 [18]. Furthermore, the H_1_R is predominantly expressed in VZ cells (Figure S5), where NSC resides [43,44]. Hence, the increased FOXP2 and the reduction of Nestin^+^ cells in the Db group, and the effect of Chlo in the Db + Chlo group suggest that the H_1_R pathway participates in the increased cortical neurogenesis, probably by promoting FOXP2 nuclear transport and NSC transition to IPs. Therefore, we propose that an increased expression and activation of classical PKCs in the dorsal telencephalon of Db pregnant rat embryos’, probably due to enhanced H_1_R activity, might promote FOXP2 nuclear transportation through phosphorylation.

Indeed, our results support the mechanism proposed. It is important to note that in the Ctl group, in addition to the expected nuclear localization of FOXP2, a cytoplasmic mark was also detected. It has been postulated that FOXP2 is exclusively localized into the nuclei. Nonetheless, their cytoplasmic localization during the rat developing cortex is supported by Western blots detection in both the cytoplasmic and nuclear protein fractions. Hence, cytoplasmic FOXP2 might be a peculiarity of the cortical development neuroepithelium since the cytoplasmic mark is not observed in ventral telencephalon (ganglionic eminence) in the Ctl group at E14 (Figure S4B). Actually, our group reported FOXP2 cytoplasmic aggregation in the motor cortex of neonates but not in 21-day-old pups [45].

Another possible explanation for cytoplasmic FOXP2 might be the presence of splice variants lacking NSL. From 19 exons, at least two (3a and 3b) generate splice variants [46]. In addition, Bruce and Margolis (2002) reported the presence of additional 5′ exons, internal exons, and alternate splice variants of humans and mice expressed in the lung and brain [46]. Unfortunately, this has not been further studied, although, in some reports, a cytoplasmic FOXP2 mark can be observed in the postnatal brain (for example, [47,48]).

Another circumstance that might contribute to FOXP2 cytoplasmic aggregation could be a high rate of protein degradation in specific brain areas during rat development, which could also explain lower molecular weight bands reported in our Western blots. Furthermore, changes in phosphorylation rates may also contribute to its subcellular localization. Therefore, studying all these molecular phenomena during development, between species, and under pathological conditions sound attractive, as cytoplasmic FOXP2 may have additional functions during brain development other than as a transcriptional factor.

The Chlo effect on the FOXP2 nuclear content in the Db group suggests that the H_1_R signaling pathway is involved in their nuclear transport. Furthermore, since FOXP2 and Ki67 are co-expressed in the VZ, an increased RG transition to IPs might occur in the DB group. However, we found lesser IPs and lower TBR2 expression in the DB group, suggesting that direct neurogenesis could take place. Direct neurogenesis arises directly from radial glia in the VZ and indirectly from IPs in the SVZ [49,50]. The above is supported by the effect observed in the Db + Chlo group on TBR2.

Chlo treatment in the Db group may also dysregulate cell proliferation and migration, as thinner neuroepithelia were observed in the Chlo + Db group. Indeed, histamine affects cortical NSC proliferation and migration at E14 [22]. Further evidence of impaired migration in the Db and Chlo + Db groups are the altered Nestin mark and the presence of IPs (TBR2^+^-cells) in the VZ. Interestingly, lower cortical proliferation has been previously reported in mouse (E11.5) NSC in vivo and in vitro under a high glucose environment [16].

To our knowledge, there is no evidence that phosphorylation regulates FOXP2 nuclear transport. However, FoxO3A, FoxO4, and FoxM1 translocate to the nuclei by phosphorylation [51,52,53]. Furthermore, the bioinformatic analysis revealed four potential sites in the FOXP2 Forkhead domain (S516, S580, T532, and T542) that might be responsible for higher FOXP2 nuclear localization in the Db group and specially S580 since it is located within NSL2. Therefore, it will be necessary to validate the role of these amino acid residues in FOXP2 subcellular localization.

Mizutani et al. (2007) studied the FOXP2 subcellular distribution in mutant mice, demonstrating that eliminating the two NLS domains or mutating one NLS abolished and decreased their nuclear localization. They also showed that substituting the basic amino acids in the NLS2 with alanine (KRR-AAA) significantly reduced nuclear FOXP2 [54]. Furthermore, our bioinformatic analysis showed that replacing the basic amino acids in the NLS2 with alanine decreased the phosphorylation score in the S^580^ (Table S4), suggesting that it is a plausible target for FOXP2 mutation and nuclear translocation studies.

Another residue essential for human FOXP2 subcellular localization is R553, its mutation in the mice sequence (R^552^H; NP_683698, according to [54]) correlated with FOXP2 cytoplasmic localization. Interestingly, in humans, this mutation is associated with speech/language disorders [9,54]. In the rat, the human R553 corresponds to the amino acid 531, which is located before T532, a putative PKC phosphorylation site. However, after analyzing the phosphorylation score, the RSA, and the disorder level of T532 of R531H, no changes were obtained in the in silico (Table S4).

The data obtained in the Mizutani study and the results presented here suggested that the S580 is a plausible site of PKC regulation to increase FOXP2 nuclear translocation in embryos of diabetic rats.

Our result on classical PKCs, particularly PKCα, suggests a possible regulation in FOXP2 transport to the nuclei and control of gene expression related to the NSC cell cycle. Interestingly, PKCs negatively and positively regulate cell cycle progression depending on the context, implying a high degree of complexity with effects involving multiple cell cycle regulatory molecules, such as cyclins, cyclin-dependent kinases, and cyclin inhibitors [55]. Furthermore, the cell cycle length is involved in NSC self-renew and differentiation. Indeed, changes in the G1 affect auto-renew and neurogenesis in a way that G1 lengthening triggers neurogenesis [56].

The antagonistic effect of PKCα on the cell cycle, even in the same cell phenotype, depends on the level of the retinoic acid [55]. Interestingly, changes in this molecule have been reported in E9-E16 embryos of diabetic rats [57,58]. So, we expected that the activity and function of PKCα might be altered in embryos from the Db group. Furthermore, FOXP2 promotes neural differentiation by interacting with retinoic acid signaling [59,60].

The increased PCKα activity in the Db groups and the reduction in the Db + Chlo group support the idea that higher nuclear FOXP2 might be due to phosphorylation and H_1_R activation. However, further studies are needed to determine the amino acid sequence involved.

Moreover, histamine increases asymmetric cell division of cortical NSC to promote neurogenesis in vitro [20]. Asymmetric cell division results from increased G1 length, which underlies neurogenesis [61]. Hence, we speculate that an increased H_1_R level in the dorsal telencephalon of embryos exposed to high glucose could alter the cell cycle through αPKC and FOXP2. However, further studies are needed to elucidate the effect of high glucose on the cell cycle and FOXP2 gene targets.

All these data suggest critical abnormalities in the cortical function triggered by a hyperglycemic environment during early embryo development, where oxidative stress and chronic inflammation contribute. Indeed, oxidative damage and inflammation are factors implicated in the etiology of autism spectrum disorder [62,63], schizophrenia [64], and neurological disorders related to maternal diabetes [65,66]. Furthermore, long-term cortical effects of maternal diabetes in the rat’s offspring involve impaired cortical cytoarchitecture, cell polarity, and reduced excitability of deep-layer cortical neurons [45].

**Figure 8:**
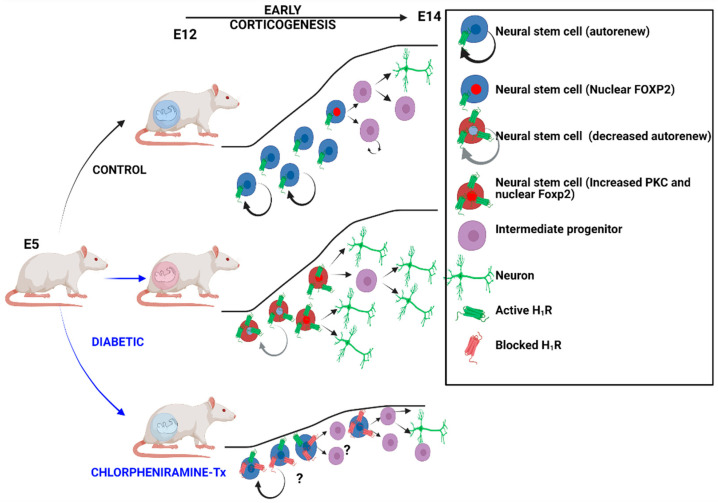
Scheme of the impaired neurogenesis of embryos from diabetes diabetic rats and the effect of H_1_R blocking. Data obtained in the present study and Solís et al., 2017. Created with BioRender.com (5 August 2022).

Overall, our study suggests that increased neurogenesis during early corticogenesis in embryos exposed to high glucose is associated with higher FOXP2 nuclear translocation due to increased phosphorylation by PKCα as a result of higher H_1_R activity, leads to a reduction of NSC by increased asymmetric cell division. However, further studies are needed to establish if directly PKCα phosphorylation is responsible for higher FOXP2 nuclear translocation, the amino acid residue involved, and the changes in FOXP2 target genes in cortical NSC in high glucose exposed embryos (Figure 8).

## Data Availability

Not applicable.

## Supplementary Materials

The following supporting information can be downloaded at: https://www.mdpi.com/article/10.3390/cells12030510/s1, Table S1. Primer sequences, aligning temperatures, and size expected in the PCR reactions; Table S2. Antibodies used in western blot and immunofluorescence assays; Table S3. Secondary antibodies information; Table S4. Putative PKC phosphorylation sites for FoxP2; Figure S1. Working scheme and embryo morphology and viability; Figure S2. Neuron markers expression in the cortical neuroepithelium at 14-day-old embryos; Figure S3. Single chlorpheniramine treatment alone does not affect neuronal markers and nuclear FOXP2; Figure S4. FOXP2 subcellular localization in the E14 ventral telencephalon. Figure S5. H_1_R immunohistofluorescence in 12-day-old cortical neuroepithelium of control and diabetic groups.
