# Supplementary material for: Increased Nuclear FOXP2 Is Related to Reduced Neural Stem Cell Number and Increased Neurogenesis in the Dorsal Telencephalon of Embryos of Diabetic Rats through Histamine H1 Receptors
Source: Cells. 2023 Feb 3;12(3):510. doi: 10.3390/cells12030510 (PMC9914739; doi:10.3390/cells12030510)
Supplement: Supplementary file 1 [file cells-12-00510-s001.zip › cells-2125852-supplementary.pdf]

cells

MDPI

Article

Increased Nuclear FOXP2 Is Related to Reduced Neural Stem Cell Number and Increased Neurogenesis in the Dorsal Telencephalon of Embryos of Diabetic Rats Through Histamine H1 Receptors

Diana Sarahi De la Merced-García [1], Ángel Sánchez-Barrera [2], Juan Hernández-Yonca [1], Ismael Mancilla [3], Guadalupe García-López [1], Néstor Fabián Díaz [1], Luis Ignacio Terrazas [3,4] and Anayansi Molina-Hernández [1,*]

[1] Departamento de Fisiología y Desarrollo Celular, Instituto Nacional de Perinatología Isidro Espinosa de los Reyes, Montes Urales 800, Miguel Hidalgo, Ciudad de Mexico 11000, Mexico
[2] Unidad de Biomedicina, Facultad de Estudios Superiores (FES)-Iztacala, Universidad Nacional Autónoma de México (UNAM), Av. de los Barrios, Los Reyes Iztacala, Tlanepantla 54090, Mexico
[3] Departamento de Infectología, Instituto Nacional de Perinatología Isidro Espinosa de los Reyes, Montes Urales 800, Miguel Hidalgo, Ciudad de Mexico 11000, Mexico
[4] Laboratorio Nacional en Salud FES-Iztacala, Universidad Nacional Autónoma de México (UNAM), Av. de los Barrios, Los Reyes Iztacala, Tlanepantla 54090, Mexico
* Correspondence: anayansimolina@gmail.com

Supplementary Material

Supplementary Tables

Table S1. Primer sequences, aligning temperatures, and size expected in the PCR reactions.

Target	Primer sequence<br>Sense (5′-3′)<br>Antisense (5′-3′)	Aligning temperature (°C)	Size bp*
FoxP2	GAA AGC GCG AGA CAC ATC G<br>GAA GCC CCC GAA CAA CAC A	63°C	222
β-III Tub	GCC AAG TTC TGG GAG GTC ATC<br>GTA GTA GAC ACT GAT GCG TTC CA	58°C	102
Map2	GAG AAG GAG GCC CAA CAC AA<br>TCT TCG AGG CTT CTT CCA GTG	66°C	132
PCKα	GCC AAG TTC TGG GAG GTC ATC<br>GTA GTA GAC ACT GAT GCG TTC CA	60°C	210
PCKβ	GAG AAG GAG GCC CAA CAC AA<br>TCT TCG AGG CTT CTT CCA GTG-	60°C	203
PCKγ	AAA AGG CCA GCT CGT GAT CC<br>CTG CTT TCC AAT GCC CCA GA	61°C	225
Gapdh	GGA CCT CAT GGC CTA CAT GG<br>CCC CTC CTG TTG TTA TGG GG	58°C	198

*PCR product sizes were confirmed by visualizing the corresponding bands after end-point PCR and agarose gel electrophoresis stained with GelRed.

Table S2. Antibodies used in western blot and immunofluorescence assays.

Antibody	RRID (catalog #)	Dilution		MW (kDa) (µg)
		WB	IHF	
Nestin	AB_11175711 (GTX39577)	1:1000	1:100	~220 (40)
FOXP2	AB_2107107 (ab16046)	1:2000	1:500	~80 (40[cy] or 20[n])
β-III TUB	AB_2210524 (MAB1637)	1:2000	1:100	~55 (60)
MAP2	AB_369978 (GTX11268)	1:5000	1:500	~280[a/b] or ~70[c] (80)
TBR2	AB_778267 (ab23345)		1:250	
Ki67	AB_10728990 (GTX84107)		1:250	
PKCα Total	AB_777294 (ab32376)	1:2500	1:1000	~77 (80)
PKCα phosphorilated (S657)	AB_2783796 (ab180848)	1:500	1:250	~77 (80)
TBP1	AB_945758 (ab51841)	1:2000		~38 (40 or 80)
GAPDH	AB_1080976 (GTX100118) AB_11174761 (GTX627408)	1:1000 1:5000		~37 (40 or 80)

*Amount of protein used for WB for cytoplasmic ([cy]) or nuclear fraction ([n]).

[a/b] and [c], corresponding to the MAP2 isoforms.

WB, Western Blot and IHF, immunohistofluorescence.

Table S3. Secondary antibodies information.

Antibody	IRRID	Dilution
IRDye 680RD	AB_10954442	1:10000
IRDye 800CW	AB_621847	1:10000
Alexa Fluor 488	AB_2576217	1:1000
Alexa Fluor 568	AB_144696	1:1000

Table S4. Putative PKC phosphorylation sites for FoxP2.

Position	Amino acid	Sequence	Score GPS 3.0	Score NetPhos 3.1	RSA %	Disorder %
5	S	***MMQESATETISN	0.296	0.724	80	100
7	S	*MMQESATETISNSS	0.418	---	80	100
9	T	MQESATETISNSSMN	0.516	0.493	80	100
11	S	ESATETISNSSMNQN	0.401	---	78	100
13	S	ATETISNSSMNQNGM	0.292	---	79	100
14	S	TETISNSSMNQNGMS	0.309	0.731	79	100
24	S	QNGMSTLSSQLDAGS	0.253	---	77	100
71	S	QQQTSGLKS	---	0.586	76	98
79	S	GLKSPKSSDKQRPLQ	0.476	0.992	78	100
292	S	DLTTNNSSSTTSSTT	0.248	0.596	77	100
294	T	TTNNSSSTTSSTTSK	0.253	0.746	75	100
297	S	STTSSTTSK	---	0.673	76	100
298	T	TTSSTTSKA	---	0.784	76	100
299	T	SSTTSSTTSKASPPI	0.424	0.893	75	100
336	T	GASHTLYGH	---	0.593	48	87
409	S	HMRPSEPKP	---	0.727	85	98
414	S	RPSEPKPSPKPLNLV	0.249	0.99	78	99
425	T	VSSVTMSKN	---	0.683	75	100
433	T	NMLETSPQS	---	0.719	77	100
443	T	PQTPTTPTA	---	0.524	79	100
446	T	PTTPTAPVT	---	0.598	79	100
463	S	ITPASVPNV	---	0.552	69	92
516	S	IMESSDRQL	---	0.518	64	1
532	T	WFTRTFAYF	---	0.618	37	0
		WFTHTFAYF	---	0.618	37	0
542	T	RNAATWKNA	---	0.842	35	1
580	S	VEYQKRRSQKITGSP	0.368	0.997	76	33
		VEYQAAASQKITGSP	---	0.794	77	29
686	T	MSLVTTANH	---	0.609	64	98
706	S	EIEEEPLSEDLE***	0.275	---	77	100

Data was obtained with GPS 3.0-Species Specific (*R. norvegicus)* [1] and NetPhos-3.1 [2]. The percentage of relative Surface accessibility and disorder were obtained by NetSurfP-2.0 [3].

NLS2 mutation of "KRR" by "AAA" [4]

Mutation of WFTR[531]TFAYF to WFTH[531]TFAYF 548 corresponds to the R553H human mutation [4].

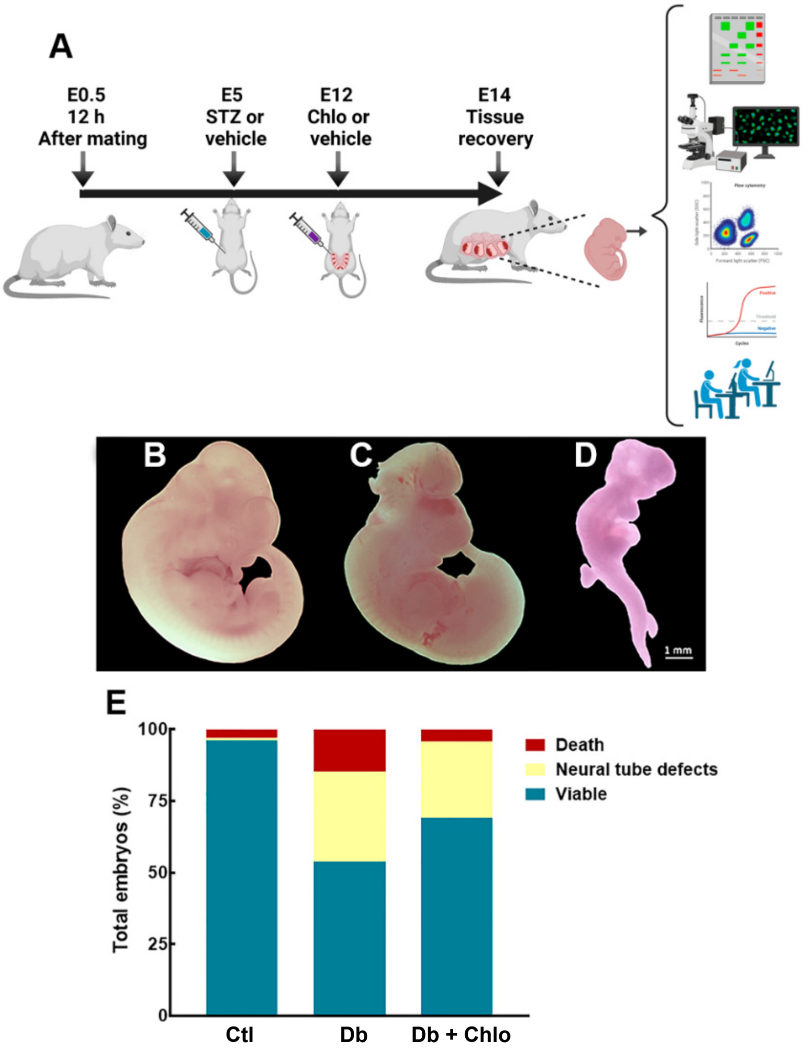

Figure S1. Working scheme and embryo morphology and viability. A) The scheme represents time point manipulation of pregnant rats, embryo recovery, and the methods used (from top to bottom: Western blot, immunofluorescence, flow cytometry, qRT-PCR, and bioinformatics analysis). E = embryo day. Created with BioRender.com (5 August 2022). **B-D)** Representative images of 14-day-old embryos obtained of diabetic rats showing viable (**B**, included in the study), neural tube defect (**C**, excluded), and death (**D**, excluded) embryos. **E)** Percentage of viable (blue), neural tube defect (yellow), and death (red) embryos obtained from control (Ctl), diabetic (Db), and diabetic chlorpheniramine-treated (Db+Chlo) pregnant rats. Data are expressed as a percentage of total embryos. n = 16 pregnant rats per group.

.

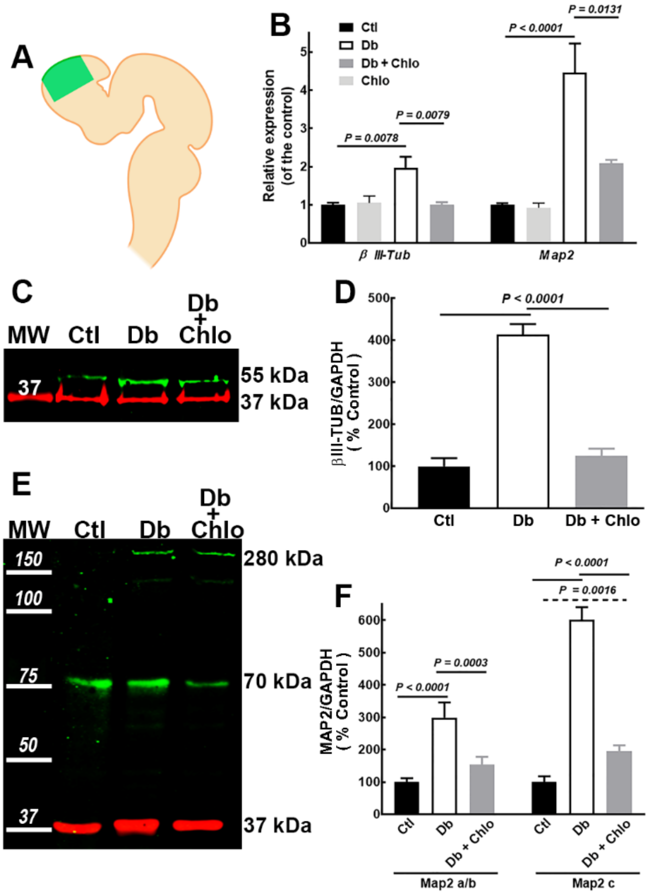

Figure S2. Neuron markers expression in the cortical neuroepithelium at 14-day-old embryos. A) Image showing the E14 neural tube. In green is highlighted the dorsal telencephalon dissected for qRT-PCR and Western blot analysis. Created with BioRender.com (1 January 2023). **B)** *βIII-Tubulin* (*βIII-Tub*) and *Map2* mRNAs relative expression in E14 dorsal telencephalon of embryos from control (Ctl), Chlorpheniramine-treated, diabetic (Db) and chlorpheniramine-treated diabetic (Db+Chlo) groups using the $2^{-\Delta\Delta CT}$ method. The *Gapdh* amplification was used as an internal control. **C** and **E)** Representatives Western Blots from E14 dorsal telencephalon from each experimental group for βIII-TUB (**C**; green, ~55 kDa) and MAP2 (**E**, green, ~70 and ~280 kDa; light and heavy isoforms, respectively). GAPDH (red, 37 kDa) was used as a loading control. **D** and **F**) Quantitative fluorometry analysis for βIII-TUB (**D**) and MAP2 (**F**). Values (means ± S.E.M., n= 4) are expressed as a percentage of the control fluorescence ratio. The two-way ANOVA was performed, followed by Tukey's multiple comparisons test. Significant *P* values are shown in the graphs. MW = molecular weight ladder.

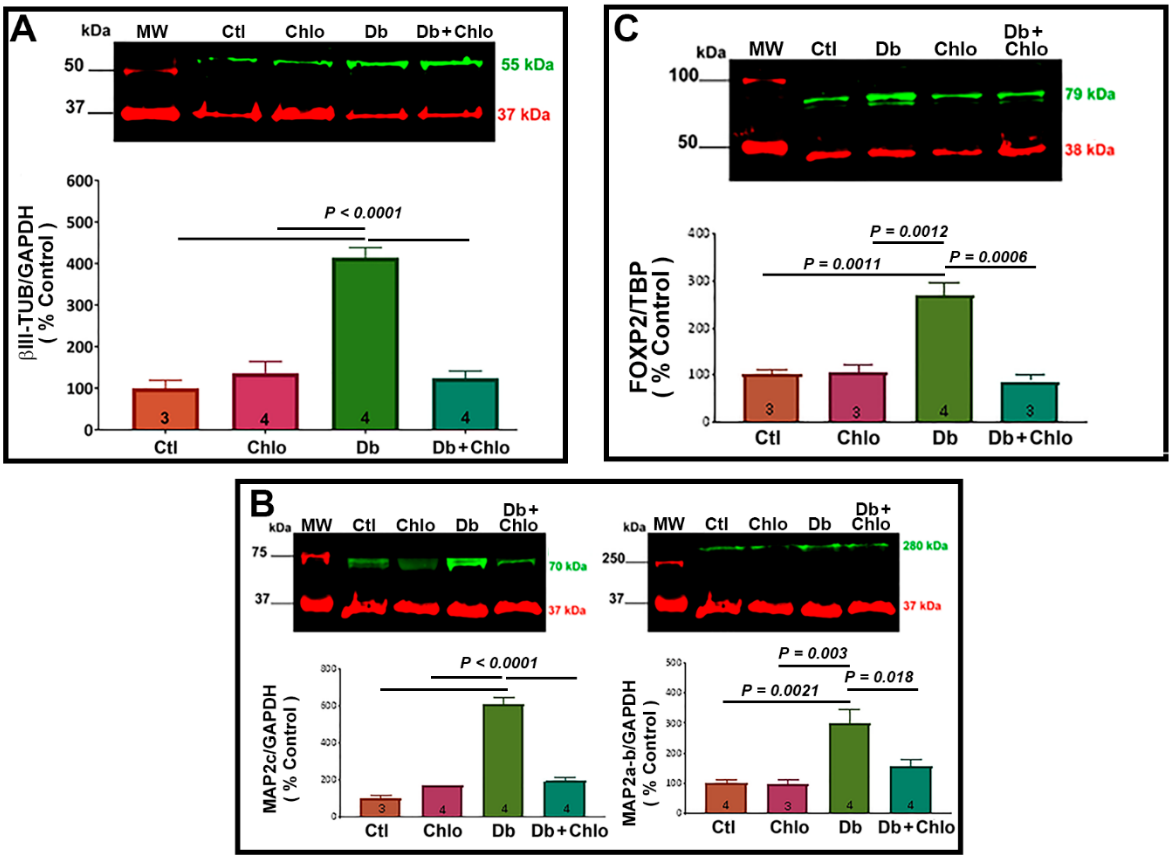

Figure S3. Chlorpheniramine alone does not affect nuclear neuronal markers and nuclear FOXP2. Top in A, B, and C) Representatives Western Blots from E14 dorsal telencephalon from control (Ctl) Chlorpheniramine-treated (Chlo), Diabetic (Db) and Chlorpheniramine-treated diabetic (Db+Chlo) groups for βIII-TUB (**A**; green, ~55 kDa), MAP2 (**B**; green, ~70 and ~280 kDa; light and heavy isoforms, respectively) and FOXP2 (**C**; green ~80 kDa). GAPDH (red, 37 kDa) and TBP (red, 38 kDa) were used as internal controls. **Down in A, B, and C)** Quantitative fluorometry analysis for βIII-TUB (**A**), MAP2 (**B**), and FOXP2 (**C**). Values (means ± S.E.M., n= are in each bar in the graphs) are expressed as a percentage of the control fluorescence ratio. The two-way ANOVA was performed, followed by Tukey's multiple comparisons test. Significant *P* values are shown in the graphs. MW = molecular weight ladder.

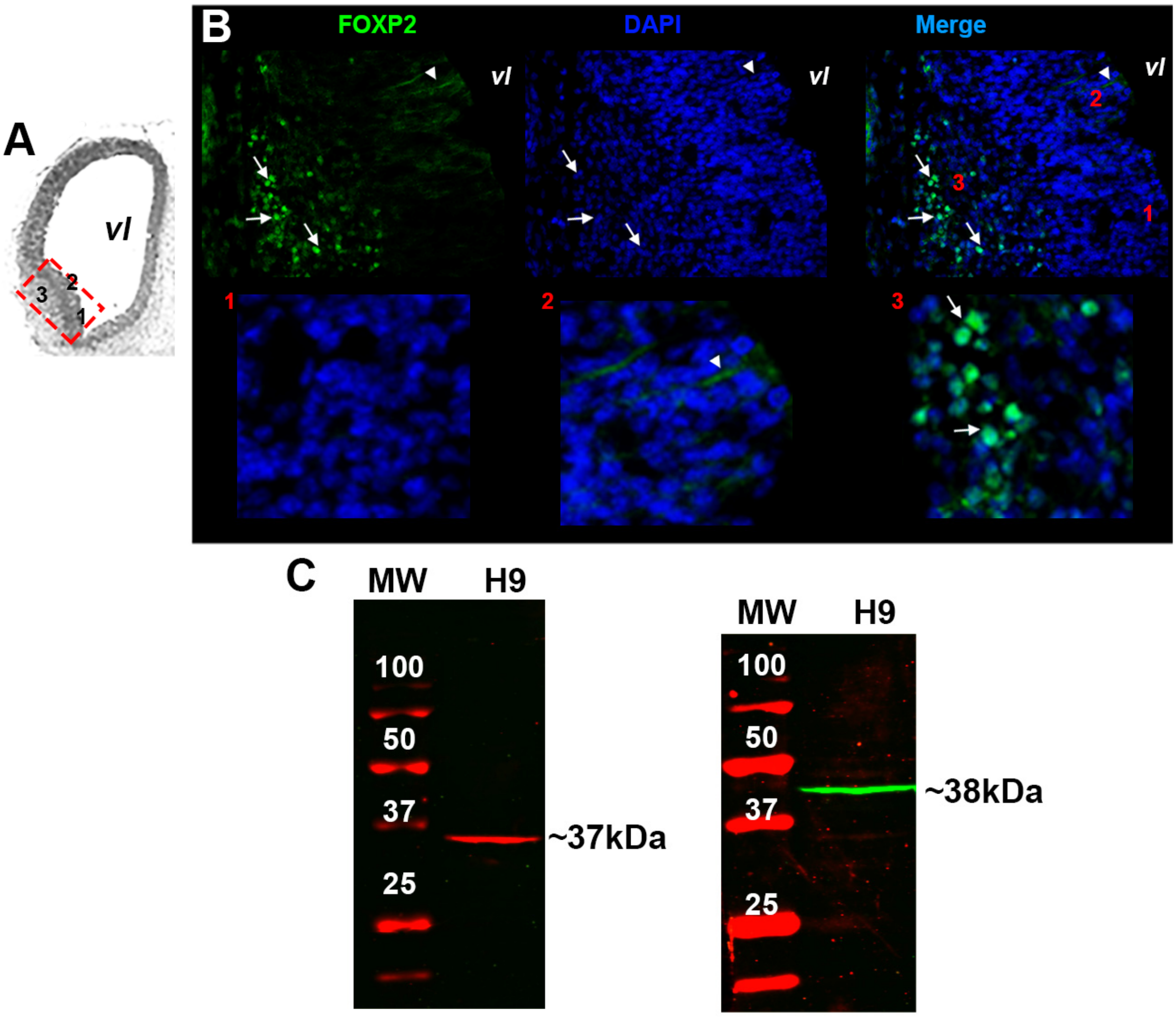

Figure S4. FOXP2 subcellular localization in the E14 ventral telencephalon. A) Hematoxylin-eosin-stained coronal section of E14 telencephalon used for the immunodetection in **B**. Numbers in black are 1 and 2 = pallidal neuroepithelium and 3 = subventricular zone of the pallidal neuroepithelium [5]. **B) Up**: from left to right: representative micrographs (20×) of the immunofluorescence of FOXP2 (green) and the nuclei stained with DAPI (blue) per channel and merged channels in the ventral telencephalon. **Down:** from left to right are electronic zooms (300%) from the corresponding areas of the red numbers in red of the merged image in the upper panel. Numbers in red are the same as in **A.** Arrows are the FOXP2 mark in the nuclei, and arrowheads are cytoplasmic, which is absent in most ventral parts of the ventral telencephalon. **C)** FOXP2 negative control Western Blot of cytoplasmic (left, green) and nuclear (right, red) protein extracts obtained from the pluripotent cell line H9. GAPDH (in red on the left, ~37 kDa) and TBP1 (in green on the right, ~38 kDa) were used as internal controls for cytoplasm and nuclear fractions, respectively.

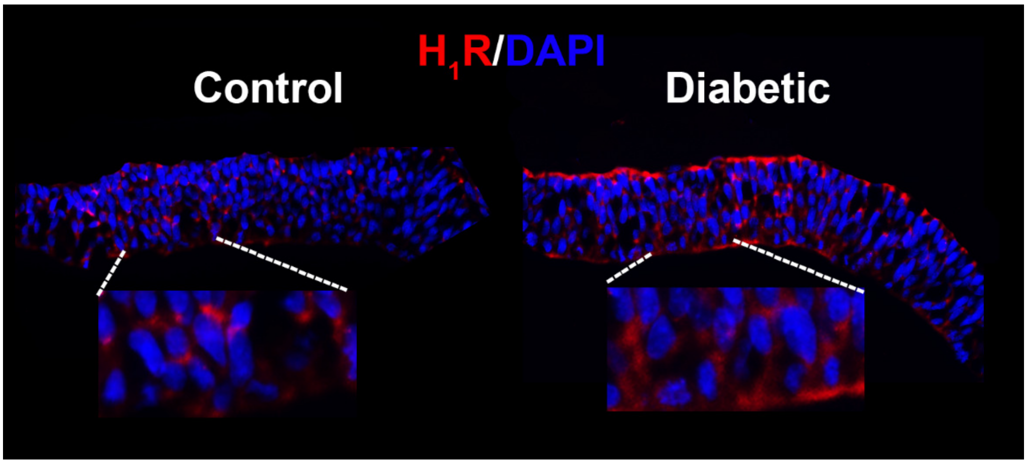

Figure S5. H1R immunohistofluorescence in the 12-day-old cortical neuroepithelium of control and diabetic groups.

References

1. Xue, Y.; Liu, Z.; Cao, J.; Ma, Q.; Gao, X.; Wang, Q.; Jin, C.; Zhou, Y.; Wen, L.; Ren, J. GPS 2.1: Enhanced prediction of kinase-specific phosphorylation sites with an algorithm of motif length selection. *Protein Eng. Des. Sel.* **2011**, *24*, 255–260. https://doi.org/10.1093/protein/gzq094.

2. Blom, N.; Gammeltoft, S.; Brunak, S. Sequence and structure-based prediction of eukaryotic protein phosphorylation sites. *J. Mol. Biol.* **1999**, *294*, 1351–1362. https://doi.org/10.1006/jmbi.1999.3310.

3. Klausen, M.S.; Jespersen, M.C.; Nielsen, H.; Jensen, K.K.; Jurtz, V.I.; Sonderby, C.K.; Sommer, M.O.A.; Winther, O.; Nielsen, M.; Petersen, B.; et al. NetSurfP-2.0: Improved prediction of protein structural features by integrated deep learning. *Proteins* **2019**, *87*, 520–527. https://doi.org/10.1002/prot.25674.

4. Mizutani, A.; Matsuzaki, A.; Momoi, M.Y.; Fujita, E.; Tanabe, Y.; Momoi, T. Intracellular distribution of a speech/language disorder associated FOXP2 mutant. *Biochem. Biophys. Res. Commun.* **2007**, *353*, 869–874. https://doi.org/10.1016/j.bbrc.2006.12.130.
